# A comparison of three different methods of eliciting rapid activity-dependent synaptic plasticity at the *Drosophila* NMJ

**DOI:** 10.1371/journal.pone.0260553

**Published:** 2021-11-30

**Authors:** Carolina Maldonado-Díaz, Mariam Vazquez, Bruno Marie

**Affiliations:** 1 Institute of Neurobiology, University of Puerto Rico, Medical Sciences Campus, San Juan, Puerto Rico; 2 Department of Anatomy & Neurobiology, University of Puerto Rico, Medical Sciences Campus, San Juan, Puerto Rico; Rosalind Franklin University of Medicine and Science Chicago Medical School, UNITED STATES

## Abstract

The *Drosophila* NMJ is a system of choice for investigating the mechanisms underlying the structural and functional modifications evoked during activity-dependent synaptic plasticity. Because fly genetics allows considerable versatility, many strategies can be employed to elicit this activity. Here, we compare three different stimulation methods for eliciting activity-dependent changes in structure and function at the *Drosophila* NMJ. We find that the method using patterned stimulations driven by a K+-rich solution creates robust structural modifications but reduces muscle viability, as assessed by resting potential and membrane resistance. We argue that, using this method, electrophysiological studies that consider the frequency of events, rather than their amplitude, are the only reliable studies. We contrast these results with the expression of CsChrimson channels and red-light stimulation at the NMJ, as well as with the expression of TRPA channels and temperature stimulation. With both these methods we observed reliable modifications of synaptic structures and consistent changes in electrophysiological properties. Indeed, we observed a rapid appearance of immature boutons that lack postsynaptic differentiation, and a potentiation of spontaneous neurotransmission frequency. Surprisingly, a patterned application of temperature changes alone is sufficient to provoke both structural and functional plasticity. In this context, temperature-dependent TRPA channel activation induces additional structural plasticity but no further increase in the frequency of spontaneous neurotransmission, suggesting an uncoupling of these mechanisms.

## Introduction

Synaptic plasticity is at the center of cognitive processes such as learning and memory [1, 2]. This plasticity consists of increased or decreased neuronal activity leading to changes at the synapse that will persist after the activity ceases. This phenomenon, thought to be the cellular correlate of learning and memory, is referred to as activity-dependent synaptic plasticity [3–6].

While our understanding of the mechanisms underlying and regulating this process has improved tremendously during the last decades [7–9], a lot is still unknown. To dissect the molecular mechanisms underlying activity-dependent synaptic plasticity, research has turned towards studying this phenomenon using a variety of model systems. Indeed, in addition to the *in vivo* work carried out in rodents (for review [10]) research also turned to hippocampal neurons in culture [11–15], *Caenorhabditis elegans* sensory system and neuromuscular junction [16–19], and the *Drosophila melanogaster* glutamatergic neuromuscular junction (NMJ) [20–23]. Methods to elicit activity-dependent synaptic plasticity at the fruit fly *Drosophila melanogaster* NMJ have been numerous. Patterned depolarizations of the larval NMJ were first achieved using a stimulation protocol consisting of 5 cycles of high activity in response to a 90mM KCl saline solution (High K^+^ protocol), direct nerve stimulation, and optogenetics. This early work validated acute stimulation results in order to elicit both structural/morphological and functional/electrophysiological modifications [23]. Since then, the High K^+^ protocol has been extensively used [24–28]. The High K^+^ protocol was then adapted to a shorter treatment that was sufficient to induce morphological changes in axotomized preparations [21, 22, 29]. Another way used to evoke activity-dependent plasticity was direct electrical stimulation. This has been used with different stimulation frequency and duration protocols and was capable of evoking both electrophysiological and morphological modifications at the NMJ [23, 29]. Structural plasticity was also reported after a continuous increase in motoneuron activity induced by expressing TRPA channels in motoneurons and exposing transgenic larva to TRPA-permissive temperature of 30°C for 1 hour [30]. These studies presented a variety of methods to evoke neuronal activity ranging from spaced depolarizations distributed within 28 minutes to a sustained depolarization of 60 minutes. Most studies have employed the High K+ activity-dependent synaptic plasticity induction protocol that involves the dissection of the larva and the patterned synchronous stimulation of the pre- and the postsynaptic compartments. Different versions with varying timescales of stimulation and rest periods have been shown to promote structural plasticity at the NMJ.

However, several questions remain. Is structural plasticity invariably coupled to functional plasticity? Is characterization of functional modifications hampered by a possible detrimental effect of the stimulation itself? What is the optimal induction protocol to study activity-dependent synaptic plasticity at the *Drosophila* NMJ? Furthermore, do all patterns of increased activity evoke the same physiological response at the NMJ?

Here we investigate and compare three different ways to elicit activity-dependent synaptic plasticity at the *Drosophila* NMJ and describe the morphological and electrophysiological changes after each of these treatments. At every step, we discuss the benefits and disadvantages of each method. We first evoke activity-dependent synaptic plasticity using a patterned High K+ stimulation protocol established previously [23], and show that this treatment provokes robust morphological changes but is detrimental to the physiological state of the muscle cell, rendering the characterization of physiological modifications difficult. We then use an optogenetics method to evoke activity-dependent synaptic plasticity. Although optogenetics has been used at the NMJ before [23, 31, 32], we provide the first evidence for using CsChrimson channels [33] to elicit activity-dependent synaptic plasticity at the NMJ using a patterned red light stimulation. We find this method very efficient, permitting both morphological and electrophysiological characterization. We finally detail the use of transgenic animals expressing TRPA1 cationic channels [34–36]. In this case, we use different temperature changes and different patterns to allow activity-dependent synaptic plasticity. Surprisingly, we find that temperature changes alone, in the absence of TRPA1 channel expression, can evoke morphological and electrophysiological alterations in the synapses. Patterned activation of temperature-driven TRPA1 can provoke additional morphological changes but no additional electrophysiological modifications, suggesting a possible uncoupling between morphological and functional changes.

## Materials and methods

### Fly stocks

The genetic strain *w*^*1*^ was used as wildtype control (Bloomington Drosophila Stock Center [BDSC] stock #145) for experiments using the High K^+^ approach. For these experiments we analyzed both male and female larvae.

We used the Gal4/UAS system [37] for ectopic expression of CsChrimson and TRPA1 constructs. Transgene constructs used for optogenetic experiments include: UAS-CsChrimson on the X chromosome (w^1118^, P[20XUAS-IVS-CsChrimson.mVenus]attP18; BDSC stock #55134) and the D42gal4 motoneuron driver (w*; P[GawB]D42, BDSC stock #8816). Genetic controls were heterozygous for the D42gal4 motoneuron driver insertion lacking the UAS CsChrimson construct.

For temperature experiments, we used the UAS-TRPA1 insertion on the 2^nd^ chromosome (w*; P[UASTrpA1(B).K]attP16, BDSC stock #26263). Genetic controls were heterozygous for the D42gal4 motoneuron driver insertion lacking the UAS TrpA1 construct.

### Rearing methods

#### General rearing

All larvae were reared in standard Drosophila cornmeal media at 25°C, except when indicated otherwise. All larvae were reared in Jazz-mix Drosophila food (Fisher Scientific product number: AS153), prepared as instructed by the manufacturer.

#### Rearing for optogenetic experiments

Larvae were reared in 400μM all-trans retinal food at 25°C, fully protected from the light by covering vials with aluminum foil. All-trans retinal (Toronto Research Chemicals product number: R240000) was initially diluted to 100mM in 95% ethanol. All-trans retinal was then added to the freshly made Jazz-mix food, only when the food temperature dropped below 57°C, for a final concentration of 400μM. The food was then dispatched in individual vials (protected from the light). Importantly, after the preparation of the all-trans retinal-containing food, a clean spatula was used to break down the solidified food within each individual vial to make the food on the bottom accessible for adult flies to feed on and lay their eggs. We also added 100μL of dH_2_O (to vials containing around 10mL of food) for moisture, and we dispersed the water around the inside surfaces of the food vials (by tapping closed vials against the table). Embryos expressing CsChrimson channels in motoneurons (using D42gal4 driver) were not viable when placed in standard cornmeal media without all-trans retinal, or when all-trans retinal-containing food was not well homogenized (suggesting that the expression of CsChrimson within motoneurons creates a toxic environment in the absence of light and exogenous retinal). When transferred for experimental purposes, larvae were always kept in complete darkness since the room lighting was sufficient to activate channels and produce strong muscle contractions.

#### Rearing for temperature experiments

Larvae were reared in standard *Drosophila* cornmeal media at 20°C until they reached the wandering third instar stage. They were then transferred to a thermocycler for exposure to specific temperature shifts (see below). When handled for experimental purposes, larvae were always kept at room temperature around 21°C.

### Stimulation methods and preparations for Immunohistochemistry and electrophysiology

#### High K+ activity-dependent plasticity stimulation protocol

We carried out a protocol adapted from previously published methods [23, 27]. Five spaced depolarizations were performed on semi-intact third instar larvae by the bath application of a modified haemolymph-like HL3 saline with high K^+^ and Ca^2+^ concentrations (70mM NaCl, 10 mM NaHCO_3_, 115 mM sucrose, 5 mM trehalose, 5 mM HEPES, 10 mM MgCl_2_, 90 mM KCl and 1.5 mM CaCl_2_) for stimulation cycles, while rest periods consisted of application of HL3 saline containing low K^+^ and Ca^2+^ concentrations (70mM NaCl, 10 mM NaHCO_3_, 115 mM sucrose, 5 mM trehalose, 5 mM HEPES, 10 mM MgCl_2_, 5 mM KCl and 0.1 mM CaCl_2_). The first three stimulations are composed of 2-minute pulses followed by 15-minute rest periods. The fourth stimulation is composed of a 4-minute pulse followed by a 15-minute rest, and a fifth and final stimulation is composed of a 6-minute stimulation followed by a 15-minute rest. Larval preparations were then stretched to complete dissection prior to immunohistochemical analysis. For electrophysiology, larvae were gently stretched, the central nervous system (CNS) was removed, and the body was then placed on an electrophysiology rig to acquire intracellular electrophysiological recordings.

#### Optogenetic activity-dependent plasticity stimulation protocol

We adapted the optogenetics method from [23]. Five spaced depolarizations were performed on intact third instar larvae by exposing transgenic larvae expressing CsChrimson channels in motoneurons to red light pulses. Chrimson is a channelrhodopsin that is activated by red light. Upon exposure to red light these channels allow sudden ion influx to motoneurons [38, 39]. Red light pulses were delivered by placing larvae in a 617nm LEDs arena (Red-Orange LUXEON Rebel LED– 122 lm; Luxeon Star LEDs, Brantford, Canada). By following a specific light pattern protocol encoded in MatLab, we achieved patterned depolarizations and elicited activity-dependent plasticity at the NMJ. All pulses consisted of a 5-minute stimulation followed by 15-minute rest periods for a total of 100-minutes per protocol. Within each 5 minutes of stimulation, larvae were exposed to 60 rapid pulses of 2 seconds of lights on and 3 seconds of lights off. All larvae were placed in a 4-well clear polystyrene dish plate (Fisher Scientific product #144444), controls were placed in a separate well from experimental larvae. Each well contained a 1 x 1 inch Kim wipe paper with 30μL of 40% sucrose in dH_2_0. All Chrimson-expressing larvae showed instantaneous muscle contractions when exposed to the light. We monitored consistent body wall muscle contractions during the “lights on” periods throughout the experimental procedure. Control larvae carried the same genetic modifications as experimental larvae but lacked the genetic construct to express Chrimson channels in motoneurons. Control larvae did not show any behavioral response to the red-light pulses. At the end of the last rest period larvae were dissected under a dissecting microscope using a blue LED light bulb for illumination (blue light produced subtle body wall muscle contraction that did not interfere with dissection, we avoided white light illumination as it resulted in strong and drastic body wall muscle contractions). For immunohistochemistry analysis, when dissection was completed, CNS still in place, all lights were turned off and larval preparations were fixed under minimum light exposure with 4% paraformaldehyde for 15 minutes at room temperature. For electrophysiology experiments, larvae were gently stretched, the CNS was removed, and the body was placed on an electrophysiology rig under a low intensity white light that did not elicit muscle contraction.

#### Temperature controlled activity-dependent plasticity stimulation protocol

Intact third instar larvae were exposed to temperature shifts controlled by a thermocycler (Eppendorf Mastercycler personal, model 5332) [40, 41], to activate genetically encoded TRPA1 channels expressed in motoneurons. Pulses were consistent with the stimulation time used during the High K+ stimulation paradigm. The first three stimulations were composed of a 2-minute high-temperature exposure, followed by a 15-minute rest period at a temperature below 24°C to avoid the activation of TRPA1 channels. The fourth stimulation was composed of a 4-minute high-temperature exposure, followed by a 15-minute rest, and a fifth and final stimulation was composed of a 6-minute high-temperature exposure, followed by a 15-minute rest. The thermocycler settings were established as: 1. T = 29.0°C or 27.0°C for 2 mins; 2. T = 21.0°C or 23.0°C for 15 mins; 3. Go to step 1, repeat 2 times; 4. T = 29.0°C or 27.0°C for 4 mins; 5. T = 21.0°C or 23.0°C for 15 mins; 6. T = 29.0°C or 27.0°C for 6 mins; 7. T = 21.0°C or 23.0°C for 15 mins; 8. Hold at 23.0°C. We used the fastest ramp speed in between different temperatures, and the lid temperature was set at 22°C throughout the protocol. All larvae were individually placed in small 0.5ml PCR tubes with a 1 x 1 inch Kimwipe paper with 30μL of dH_2_0 to provide a humid environment. Genetic controls were manipulated alongside experimental larvae in the same PCR machine. At the end of the last rest period, larvae were dissected under a dissecting microscope at room temperature (around 21°C). For immunohistochemistry analysis, when dissection was completed and the CNS was still in place, larval preparations were fixed at room temperature with 4% paraformaldehyde for 15 minutes. For electrophysiology experiments, larvae were gently stretched, the CNS was removed, and the body was then placed on an electrophysiology rig for quantal analysis.

### Immunohistochemistry

Larval preparations were fixed with 4% paraformaldehyde for 15 minutes and washed in PBT 0.1% for 1 hour. Primary antibody mouse anti-Dlg (1:20; Developmental Studies Hybridoma Bank, 4F3 anti-discs large) was applied overnight at 4°C. Larval fillets were then washed in PBT 0.1% for 1 hour. Anti-Hrp Cy3-conjugated AffiniPure goat anti-horseradish peroxidase (1:300; Jackson ImmunoResearch product #123-165-021) and secondary antibody goat anti-mouse Alexa Fluor 488-conjugated AffiniPure goat anti-mouse IgG (1:300; Jackson ImmunoResearch product #115-545-166) were incubated for 1 hour at room temperature. Then a final wash in PBT 0.1% for 1 hour was followed by mounting on a glass slide with Vectashield (Vector Labs).

### Quantification of ghost boutons

**Fig 1:**
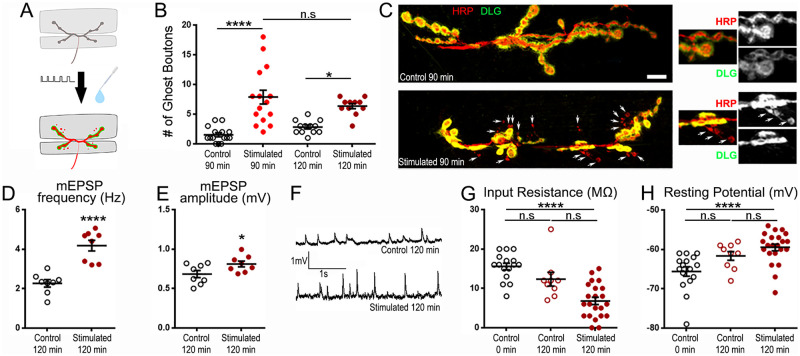
Potassium-driven stimulation evokes both structural and electrophysiological changes at the NMJ but is detrimental to muscle health. A: Schematic diagram of the NMJ undergoing patterned stimulation under the Potassium stimulation method and giving rise to *de novo* synaptic structures. B: Quantification of the average number of ghost boutons at the muscle 6/7 NMJ (segment A3) with and without stimulation. C: 2 representative NMJs at m6/7 segment A3 with immunofluorescence for a presynaptic membrane marker (red; anti-HRP) and post synaptic Discs-Large marker (green; anti-DLG). Note that arrows point out at ghost boutons showing presynaptic fluorescence but devoid of postsynaptic immunolabelling. D: Quantification of the average mEPSP frequency with and without stimulation. E: Quantification of the average mEPSP amplitude with and without stimulation. F: Representative electrophysiological recordings showing spontaneous mEPSP in control and stimulated preparations. G: Quantification of the average muscle input resistance with and without stimulation. H: Quantification of the average muscle resting potential with and without stimulation. All animals are w^1^. **** is p < 0.0001; * is p < 0.05. Kruskal-Wallis analysis with Dunn’s post-test was performed in B, G, and H. Unpaired two-tailed t-test was performed in D and E. All quantifications show SEM. Scale is 10 μm.

Identification of new synaptic structures (called *ghost boutons*, as per the previous literature) following the activity-dependent plasticity stimulation protocol was achieved by immunological staining of the NMJ, using a presynaptic (HRP) and a postsynaptic (Dlg) marker. “Ghost boutons” are newly formed synaptic boutons that lack postsynaptic differentiation; therefore, they are identified as being Hrp-positive and Dlg-negative. For each condition, control preparations were treated together with experimental preparations to account for variations in our experimental manipulations. Quantifications were performed on NMJs of muscles 6/7 on segment A3 (right and left side of the larva) and averaged across conditions. We used a Nikon Eclipse 80i microscope at a magnification of 400X to carry out ghost bouton identification. Representative images were acquired using a Nikon Eclipse Ti inverted A1R laser scanning confocal microscope. Images were acquired with oil immersion 40x with a digital zoom of 2X (only for Fig 1), and oil immersion 60x objective. NIS elements Advance Research 4.5 acquisition and analysis software was used for image acquisition. Fiji (Image J) image processing software was used for conversion of stacks into a single Maximum Intensity Projection, then converted to RGB color TIFF image file format.

### Electrophysiology

Intracellular electrophysiological recordings were used to assess modifications in synaptic function following the activity-dependent synaptic plasticity protocol. Recordings were performed on muscle 6, segment A3 (right and left side of the larva), using a sharp microelectrode of borosilicate glass with a resistance of 12–20 MΩ filled with 3M KCl. All recordings presented for the quantification of functional plasticity have resting membrane potentials lower than -60 mV, and muscle input resistance above 5MΩ. Only Fig 1G and 1H include recordings with resting membrane potentials higher (more positive) than -60mV and muscle input resistance below 5 MΩ, in order to characterize the viability of the muscle after 5 pulses of depolarizations with high potassium. Data was quantified with Synaptosoft semi-automated data analysis software. Frequency and amplitude of spontaneous neurotransmission were established by measuring 100 continuous individual mEPSP events per NMJ recorded. The average mEPSP frequency and amplitude were then averaged per condition. For evoked responses, we averaged the amplitude of 20 suprathreshold evoked EPSPs for each NMJ, and then averaged all NMJs analyzed for each condition.

### Statistical treatment

We used the GraphPad Prism 6 to analyze the data presented in this manuscript. We first assessed whether data conformed to a normal distribution by performing a Shapiro-Wilk normality test. When the Shapiro–Wilk normality test was low (p < 0.05), we ran nonparametric tests. When comparing more than two different samples, we performed a Kruskal–Wallis test with a post hoc Dunn’s multiple comparisons test. When comparing two samples, a Mann-Whitney analysis was performed. When the sample distribution was normal, we ran a parametric one-way ANOVA when comparing more than two samples. The post hoc Holm-Sidak’s multiple comparisons test was used for multiple comparisons between data sets. When only two data sets were compared, we performed an unpaired, two-tailed t test. The results of these statistical treatments are shown in the graphs of the different figures, and the specific test used is described in the figure legend.

## Results

### The K^+^-rich depolarizing method elicits potent synapse remodeling while reducing muscle health

To evoke activity-dependent synaptic plasticity at the *Drosophila* NMJ, we first used a method of patterned depolarization by repeatedly applying a depolarizing solution (rich in calcium and potassium) followed by a physiological solution (low in calcium and potassium) allowing the preparation to rest (see Materials and methods section). Using this method, motoneurons can be stimulated in a way reminiscent of the stimulation received by hippocampal neurons leading to activity-dependent synaptic plasticity [42–46]. As a result of this stimulation, well-documented morphological and electrophysiological changes ensue [23–27, 47]. Indeed, *de novo* synaptic boutons are formed. They are mature after 24 hours but after 1 to 2 hrs only the presynaptic side is present, making this stage ideal to identify and quantify them (Fig 1A) [23]. Immunoreactivity revealing the presence of a presynaptic side and the absence of postsynaptic differentiation allows the identification of these boutons, termed “ghost boutons”, and is used to quantify the magnitude of activity-dependent synaptic plasticity (Fig 1B and 1C). In our hands, we see that, while there are few ghost boutons in unstimulated controls (average of 1.5 ± 0.3, Fig 1B and 1C), their numbers increase tremendously after stimulation (average of 7.9 ± 1.2, Fig 1B and 1C; p < 0.0001). Because phenomena of activity-dependent synaptic plasticity are time sensitive, we looked at 2 different times after the start of the repeated stimulation treatments; a rest of 15 min for a total treatment of 90 min and a rest of 45 min for a total procedure of 120 min. We did not see any difference between these two conditions (compare 7.9 ± 1.2 for 90 min with 6.4 ± 0.4 for 120 min in Fig 1B, p = 0.51) suggesting that 15 min of rest after the last pulse of the stimulation is enough to evoke a full activity-dependent synaptic plasticity response and that this synaptic remodeling persists. In addition to these morphological changes, modifications of electrophysiological properties also occur. Because they are measured 45 min after the last pulse of the stimulation, at the time when ghost boutons are immature, these changes are thought to be independent of *de novo* bouton formation and represent a modification in the basic properties of the original synaptic structures [23, 26]. Indeed, the frequency of miniature excitatory post synaptic potentials (mEPSPs) is increased (compare 2.3 ± 0.2 Hz at rest with 4.2 ± 0.3 Hz after stimulation; Fig 1D and 1F; p < 0.0001). In addition, after repeated stimulation, we see a small but statistically significant increase in mEPSP amplitude (0.68 ± 0.045 mV at rest and 0.81mV ± 0.037 mV after stimulation; Fig 1E; p = 0.046) that might represent an effect also described by others [23]. While the increase in mEPSP frequency is dependent on transcription, translation [26], and Wingless signaling [23], little is known about this subtle increase in mEPSP amplitude besides the fact that it is not dependent on Wingless signaling [23]. It was hypothesized to result from a variety of presynaptic modifications like the release of multiple vesicles at the same time, or an increase in vesicle size that was previously reported [48]; or postsynaptic changes like modifications in glutamate receptor function [23].

During our electrophysiological experiments, we noticed a reduction in stimulated muscles’ input resistance and a depolarization of their resting potential. Because the input resistance has been characterized as a factor influencing mEJP amplitude [49–51] and because the resting potential is typically used to assess membrane integrity after electrode penetration, we and others have defined criteria allowing recording of mEPSPs and EPSP (see Materials and methods). Surprisingly, most of the preparations after stimulation failed to pass these criteria (see S1 Table). We therefore decided to characterize the effect of K+ stimulation on muscle input resistance and resting potential which are readily quantifiable criteria for assessing muscle viability [52, 53].

Two factors could be detrimental to muscle health, the time (120 min) left exposed as a semi-intact preparation in physiological serum [54] (Material and Methods), and the repeated high-potassium depolarizations. To test the relative importance of these two conditions, we measured preparations dissected and immediately recorded (0 min), preparations dissected and left in the physiological serum for 120 min before being recorded, and preparations repeatedly stimulated for 120 min. We find that muscle health is not greatly affected in semi-intact preparations spending 120 min in physiological saline; although the resting potential appears to be slightly depolarized at 120 min there is no statistically significant difference between median values (compare -65.6 ± 1.2 mV at 0 min with -61.7 ± 1.1 mV at 120 min; Fig 1H; p = 0.17); similarly the change in input resistance is also not statistically significant (compare 15.6 ± 0.9 MΩ at 0 min with 12.3 ± 1.7 MΩ at 120 min; Fig 1G; p = 0.24). In contrast, the preparations that underwent repeated potassium stimulations show clear signs of muscle distress because their mean resting potential is significantly reduced by about 9% (-59.5 ± 0.9 mV at 120 min, Fig 1H; p < 0.0001 compared to controls at 0 min) and their average input resistance is reduced by 56% to 6.8 ± 0.9 MΩ (Fig 1G; p < 0.0001 compared to controls at 0 min). We also noted that there was no statistically significant difference between controls at 120 min and stimulated preparations at 120 min for both Input resistance (Fig 1G; p = 0.08) and resting potential (Fig 1H; p = 0.4). This suggests that it is the combination of both time and K+ stimulation that is responsible for the observed deleterious effects on Input resistance and resting potential. This does not affect the conclusions/observations we and others made on the frequency of mEPSPs; the increase in frequency after stimulation could be, if anything, underestimated. Indeed, mEPSP decreased amplitude due to the state of the muscle could mean that some mEPSPs are not counted. In addition, the quantification of mEPSP frequency and amplitude (Fig 1D–1F) were made on the subgroup of synapses presenting an input resistance greater than 5 MΩ and a resting potential more hyperpolarizing than -60 mV (see Materials and methods). Nevertheless, this provides a strong argument for the need of a less invasive form of repeated stimulation to induce activity-dependent synaptic plasticity.

### Activation of transgenically-encoded CsChrimson in motoneurons elicits synapse remodeling and allows electrophysiological recordings

**Fig 2:**
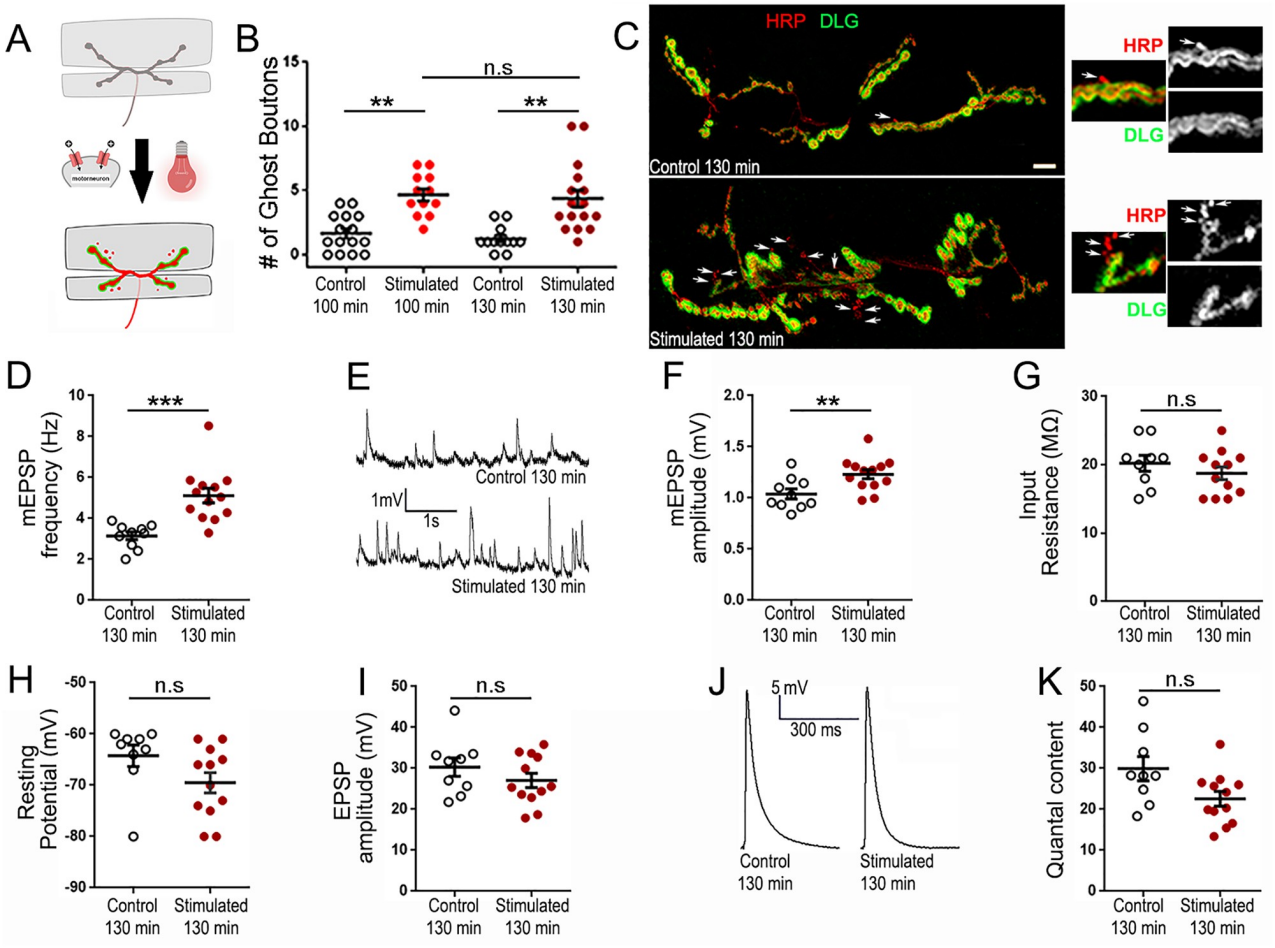
Chrimson-driven activity-dependent plasticity at the *Drosophila* NMJ. A: Schematic diagram of the NMJ undergoing optogenetic stimulation using red-light. B: Quantification of the average number of ghost boutons at the muscle 6/7 NMJ (segment A3) with and without stimulation. C: 2 representative NMJs at m6/7 segment A3 with immunofluorescence for a presynaptic membrane marker (red; anti-HRP) and post synaptic Discs-Large marker (green; anti-DLG). Arrows indicate ghost boutons showing presynaptic fluorescence but devoid of postsynaptic immunolabelling. D: Quantification of the average mEPSP frequency with and without stimulation. E: Representative electrophysiological recordings showing spontaneous mEPSPs in control and stimulated preparations. F: Quantification of the average mEPSP amplitude with and without stimulation. G: Quantification of the average muscle input resistance with and without stimulation. H: Quantification of the average muscle resting potential with and without stimulation. I: Quantification of the average evoked EPSP amplitude in unstimulated and stimulated preparations J: Representative traces of evoked EPSPs in unstimulated and stimulated preparations. K: Quantification of the average quantal content (number of vesicles released by action potential). Unstimulated animals were D42-Gal4/+ and stimulated animals were UAS-CsChrimson/+ (female) or UAS-CsChrimson/Y (male); D42-Gal4/+; *** is p < 0.001; ** is p < 0.01. Kruskal-Wallis analysis with Dunn’s post-test was performed in B. Unpaired two-tailed t-test was performed in D, F, G, I and K. Mann-Whitney analysis was performed in H. All quantifications show SEM. Scale is 10 μm.

Looking to improve the overall state of our preparations we decided to perform optogenetic stimulation. We established transgenic animals expressing the red-light gated cation channel CsChrimson [38] under the control of D42-Gal4 (driving the expression in motor and sensory neurons; [55]). Like in other optogenetics experiments [33], the opening of the CsChrimson channel leads to the depolarization of the cell in which it is expressed [38]. Chrimson is a Channelrhodopsin activated by high wavelengths of light, with the strongest response at 590nm [38], which penetrate the cuticle better than shorter wavelengths [56, 57]. When expressed in motoneurons it can elicit action potentials leading to muscle depolarization [38]. Nevertheless, to date it has not been used at the *Drosophila* NMJ to induce synaptic plasticity. To this effect, we raised these animals on all-trans retinal containing food and submitted them to a patterned light stimulation (see Materials and methods and Fig 2). We found that this treatment can provoke the appearance of ghost boutons, the morphological modifications characteristic of activity-dependent synaptic plasticity. Indeed, our unstimulated control preparations show the appearance of 1.7 ± 0.4 ghost boutons while the preparation stimulated with our optogenetic strategy showed 4.7 ± 0.5 ghost boutons (Fig 2B and 2C; p = 0.0007). These results are similar to previous observations [23, 32], where the authors used the blue-light-gated channel Channelrhodopsin-2 under the control of C380-Gal4 and OK6-Gal4 to drive optogenetic stimulation at the larval NMJ. Like us, they showed that presynaptic optogenetic stimulation induced fewer structural changes when compared to the K+-driven stimulation. This is probably because a K+ shock directly depolarizes the postsynaptic muscle. In contrast, optogenetic stimulation is only driven in a subset of neurons and thus induces activity-dependent synaptic plasticity that is the sole consequence of repeated neuronal synaptic activity. We also tested whether allowing an additional 30 min of rest would induce the formation of more ghost boutons. Indeed, in these conditions the unstimulated preparations show the presence of 1.3 ± 0.3 ghost boutons while the stimulated preparations show 4.4 ± 0.7 (Fig 2B; p = 0.0003). These data are not significantly different from the ones observed after only 15 min of rest after the last pulse (Fig 2B).

Because the main reason for a shift to optogenetics was to preserve the preparations and allow for electrophysiological recordings, we tested the input resistance and resting potential for the preparations subjected to optogenetic stimulation. We found that, in stark contrast to the K^+^-driven stimulation, there are no deleterious effects associated with the optogenetic protocols. Indeed, the mean input resistance of our unstimulated control preparations is 20.2 ± 1.2 MΩ while the preparations stimulated by optogenetics showed a comparable input resistance of 18.8 ± 0.9 MΩ (Fig 2G; p = 0.3). Importantly, there was no depolarization of muscle resting potential in stimulated preparations; stimulated preparations showed a mean resting potential of -69.5 ± 2 mV compared to controls (-64.2 ± 2.1 mV), although this difference was not statistically significant (Fig 2H; p = 0.05). These two electrophysiological characteristics illustrate that optogenetic stimulation does not affect the health of the preparation. Having achieved this, we then assessed the potentiation of mEPSP release frequency. We found that there is a 65% increase in mEPSP frequency after stimulation. Control preparations show an average frequency of 3.1 ± 0.2 Hz while the stimulated preparations show an average of 5.1 ± 0.4 Hz (Fig 2D and 2E; p = 0.0002). The amplitude of the mEPSPs is also increased after this stimulation paradigm (1 ± 0.05 mV for controls and 1.2 ± 0.04 mV after stimulation, Fig 2E and 2F; p = 0.008). The evoked EPSP amplitudes or the quantal content do not show any difference compared to controls. After stimulation, the average EPSP amplitude is 27.1 ± 1.7 mV and is not significantly different from control measurements (30.3 ± 2.3 mV; Fig 2I and 2J; p = 0.27). This is reminiscent of previous data showing that EPSP amplitude does not change upon 5 cycles of spaced depolarizations [23]. The resulting quantal content (number of vesicles released by action potential) also shows no statistically significant change. Quantal content is 29.9 ± 3 in controls and 22.6 ± 1.8 in stimulated preparations (Fig 2J and 2K; p = 0.054). In any case, we show that this optogenetic manipulation is adequate for the assessment of activity-dependent synaptic plasticity both at the morphological and electrophysiological levels.

### The effect of temperature and of temperature-driven activation of TRPA on synaptic morphology and function

Another way to manipulate neuronal activity is to use transgenic flies expressing the temperature sensitive TRPA channel in motoneurons. This warmth-activated channel can elicit depolarization in other systems. In adult *Drosophila* flies, depolarization of photoreceptor cells was achieved with genetically encoded expression of TRPA1 channels in these cells and exposure of flies to a continuous stimulus of 29°C [35]. In larval motoneurons, chronic neuronal overactivation was achieved with cell-specific TRPA1 expression and a continuous exposure to rearing temperatures of 25°C and 27°C [58]. The use of temperature as the triggering factor for motoneuron stimulation is interesting. Indeed, temperature can penetrate tissues more efficiently than light [59] which could constitute an incremental improvement compared to the optogenetics stimulation. In addition, the experimental setup is simpler and more affordable (bain-marie or thermocycler; see Materials and methods). Although TRPA-driven stimulation to promote rapid activity-dependent synaptic plasticity has been used at the *Drosophila* NMJ, it was utilized to depolarize motoneurons in one continuous stimulus of permissive temperature exposure [30].

**Fig 3:**
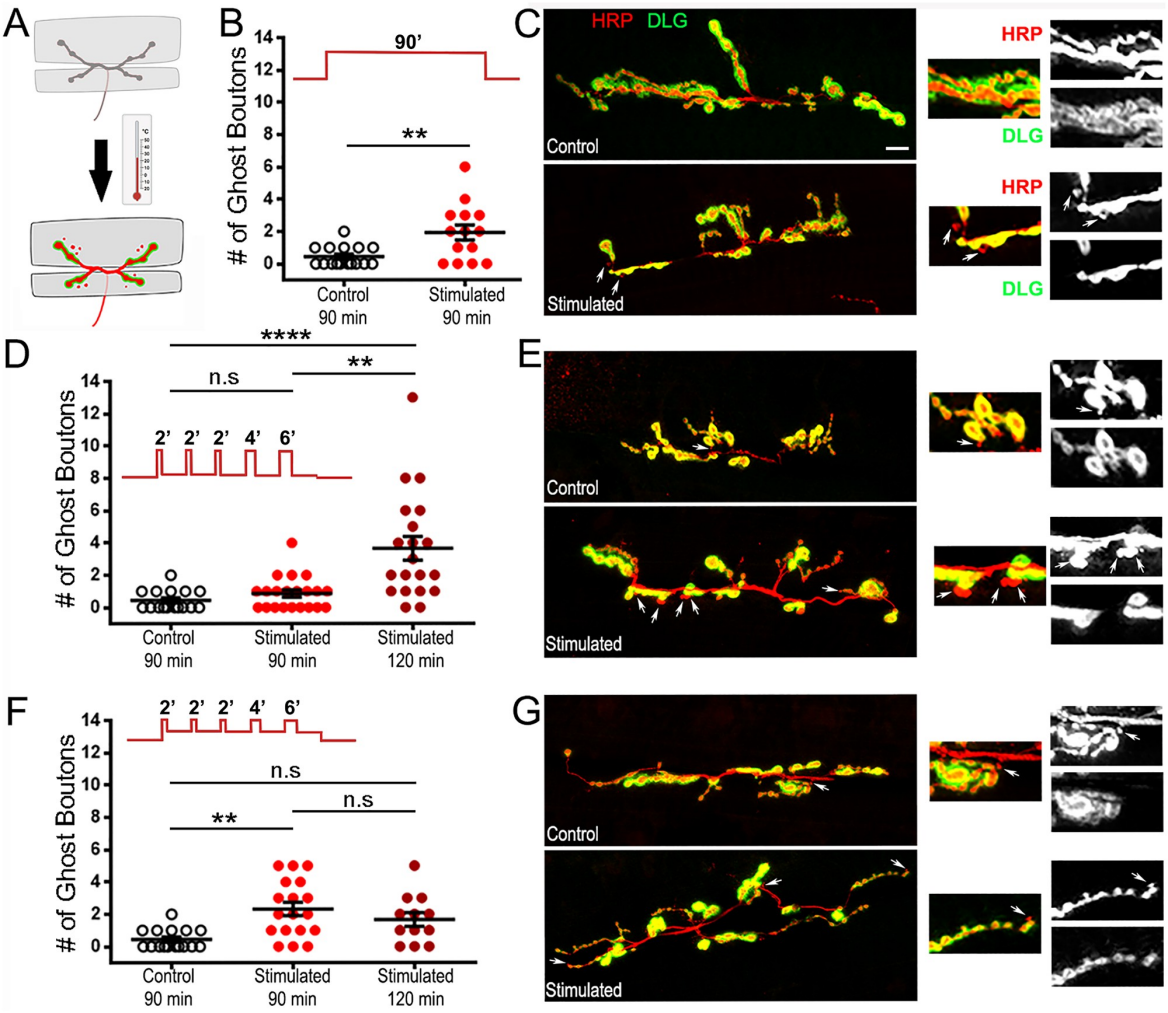
Temperature can drive structural activity-dependent synaptic plasticity. A: Schematic diagram of the NMJ submitted to a temperature stimulus. B, D, and F: Quantification of the average number of ghost boutons at the muscle 6/7 NMJ (segment A3) with and without stimulation. D and F: a different rest time was applied after the stimulation protocol and indicated on the graphs (total time of 90 min or 120 min). C, E, and G: representative NMJs at m6/7 segment A3 with immunofluorescence for a presynaptic membrane marker (red; anti-HRP) and post synaptic Disc-Large marker (green; anti-DLG). Arrows indicate ghost boutons showing presynaptic fluorescence but devoid of postsynaptic immunolabelling. B and C: A constant temperature change of 29°C was applied. D and E: Patterned steps from 21°C to 29°C were applied. F and G: Patterned steps from 23°C to 27°C were applied. All animals were D42-Gal4/+. **** is p < 0.0001; ** is p < 0.01. Mann-Whitney analysis was performed in B. Kruskal-Wallis analysis with Dunn’s post-test was performed in D and F. All quantifications show SEM. Scale is 10 μm.

Because temperature is an important factor influencing an array of behavioral [60] and physiological characteristics like gene expression [61], RNA editing [62–64], and protein activity including ion channel kinetics [65, 66], we asked whether temperature on its own could have an effect on NMJ structure. We first subjected preparations lacking TRPA channels raised at 20°C to a constant temperature of 29°C for 90 min, before assessing NMJ structure. In control preparations (kept at 20°C), ghost boutons average 0.4 ± 0.2 per synapse while after 90 min at 29°C they average 1.9 ± 0.5 (Fig 3B and 3C). This is a significant, almost 5-fold increase (p = 0.0076), strongly suggesting that a constant rise in temperature alone can provoke morphological changes at the synapse. However, our intention in this study was to evoke patterned depolarizations in motoneurons in an attempt to mimic physiological stimuli [23, 47]. We asked whether the same phenomenon could be observed if we applied a stimulation protocol consisting of 5 cycles of temperature pulses. We developed a patterned stimulation protocol using temperature pulses, based on the previously described potassium-based and optogenetic depolarization protocols (see Materials and methods). We chose 21°C as the resting temperature and gave pulses of 29°C temperature [34, 36]. We also tested whether allowing for an additional 30 min of rest could have an effect, since structural changes that arise as a consequence of activity-dependent synaptic activity are expected to promote lasting changes at the NMJ. In the experiments in which we assessed the appearance of ghost boutons in control animals 15 min after the last pulse of the stimulation (stimulated 90 min) we observed a mean of 0.9 ± 0.2 ghost boutons per synapse compared to controls (mean 0.4 ± 0.2 boutons. However, this change was not statistically significant (Fig 3D; p = 0.85). We then asked if these ghost boutons could develop after an additional 30 min of rest. To our surprise there was a large, significant difference, with an average of 3.7 ± 0.7 ghost boutons (Fig 3D and 3E; p < 0.0001). This suggests that patterned temperature stimulation of control animals is sufficient to provoke morphological changes at the synapse typical of activity-dependent synaptic plasticity.

We then wondered if this phenomenon of temperature-evoked activity-dependent synaptic plasticity in control animals would hold for smaller temperature steps. We therefore looked for the minimal temperature range that would leave the TRPA channel inactive at one extreme and trigger its activation at the other. Excitatory junction potentials were identified at the larval NMJ expressing TRPA1 channels with temperatures above 25°C [34], and tonic spikes were identified in water baths over 26°C, while 23–24°C temperatures did not generate action potentials [36]. We therefore decided to apply 23°C as a resting temperature and 27°C as a stimulating temperature to control animals. In these conditions, the average number of ghost boutons was 2.3 ± 0.4 (Stimulated 90 min; Fig 3F and 3G; p = 0.0012) and after allowing for an additional 30 min of rest 1.7 ± 0.4 (p = 0.06), whereas controls showed only 0.4 ± 0.2 ghost boutons. This shows that this treatment too can elicit a morphological activity-dependent synaptic plasticity response. Interestingly, because we saw an effect after 90 min with the 23–27°C protocol and not with the 21–29°C protocol, it could mean that 23°C is enough to provoke a temperature driven stimulation during rest periods while 21°C is not. Taken together our data show that higher temperatures applied continuously or in pulses affect the NMJ and provoke morphological effects typical of activity-dependent plasticity.

**Fig 4:**
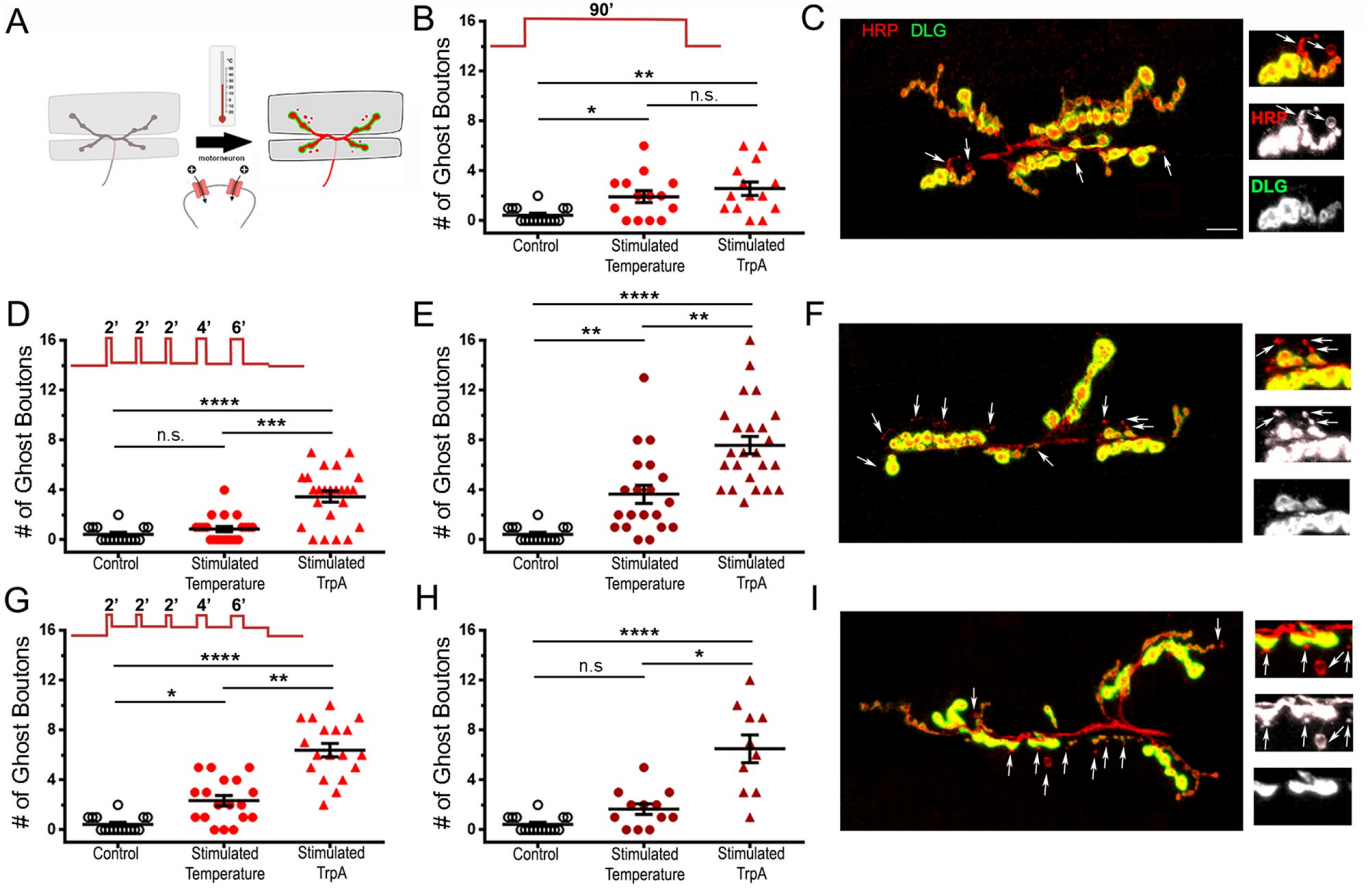
TRPA-driven activity-dependent structural plasticity at the *Drosophila* NMJ. A: Schematic diagram of the NMJ submitted to a temperature stimulus that allows the entry of cations through the TRPA channels. B, D, E, G, and H: Quantification of the average number of ghost boutons at the muscle 6/7 NMJ (segment A3) with and without stimulation. D and G: Preparations were given 15 min rest time (total procedure time of 90 min). E and H: a rest time of 45 min was applied after the stimulation protocol (total time of 120 min). C, F, and I: representative stimulated NMJs from animals expressing the TRPA transgene at muscle 6/7 segment A3 with immunofluorescence for a presynaptic membrane marker (red; anti-HRP) and post synaptic Disc-Large marker (green; anti-DLG). Arrows indicate ghost boutons showing presynaptic fluorescence but devoid of postsynaptic immunolabelling. B and C: A constant temperature change of 29°C was applied. D-F: Patterned steps from 21°C to 29°C were applied. G-I: Patterned steps from 23°C to 27°C were applied. B, D and G: data was collected 90min after the start of the protocol. E and H: data was collected 120min after the start of the protocol. Animals were D42-Gal4/+ (Control and Stimulated temperature) or UAS-TrpA/+; D42-Gal4/+ (Stimulated TrpA). **** is p < 0.0001; *** is p < 0.001; ** is p < 0.01 and * is p < 0.05. Kruskal-Wallis analysis with Dunn’s post-test was performed in all the graphs. All quantifications show SEM. Scale is 10 μm.

We then asked whether transgenic animals expressing the TRPA construct and submitted to the same stimuli could show additional changes in synaptic morphology. We first used the continuous temperature protocol and showed that there is no increased effect due to the presumed additional TRPA stimulation (Fig 4B and 4C). This interesting result suggests that the effects observed under these conditions depend on temperature and not TRPA-driven depolarization. This might be because TRPA is more sensitive to a change in temperature than to its absolute value. We therefore asked whether the patterned stimulation protocols could show increased morphological modifications. Indeed, in all the 4 protocols that we tested (pulses going from 21°C to 29°C and 23°C to 27°C; 90 and 120 min after the start of the stimulation; Fig 4D–4I) we observed an increase in ghost bouton formation when we compared temperature stimulation alone to temperature-activated TRPA animals. For stimulation using pulses from 21°C to 29°C, we observed 0.9 ± 0.2 (after 90 min) and 3.7 ± 0.7 (after 120 min) ghost boutons with temperature alone and 3.7 ± 0.7 (after 90 min; p = 0.001) and 7.6 ± 0.7 (after 120 min; p = 0.007) ghost boutons in animals expressing TRPA. Similarly, using pulses from 23°C to 27°C induced 2.3 ± 0.4 (after 90 min) and 1.7 ± 0.4 (after 120 min) ghost boutons with temperature alone and 6.4 ± 0.5 (after 90 min; p = 0.0012) and 6.5 ± 1.1 (after 120 min; p = 0.025) ghost boutons in animals expressing TRPA. This suggests that TRPA activation can efficiently depolarize the motoneurons and create morphological modifications as a consequence of activity-dependent synaptic plasticity. It is worth keeping in mind that temperature affects the entire organism, including the postsynaptic muscle fiber. Hence the effects observed by depolarizing a neuronally-expressed TRPA channel are likely a composite of the effect originating from the presynaptic TRPA-driven Ca^2+^ influx and a more general temperature effect.

**Fig 5:**
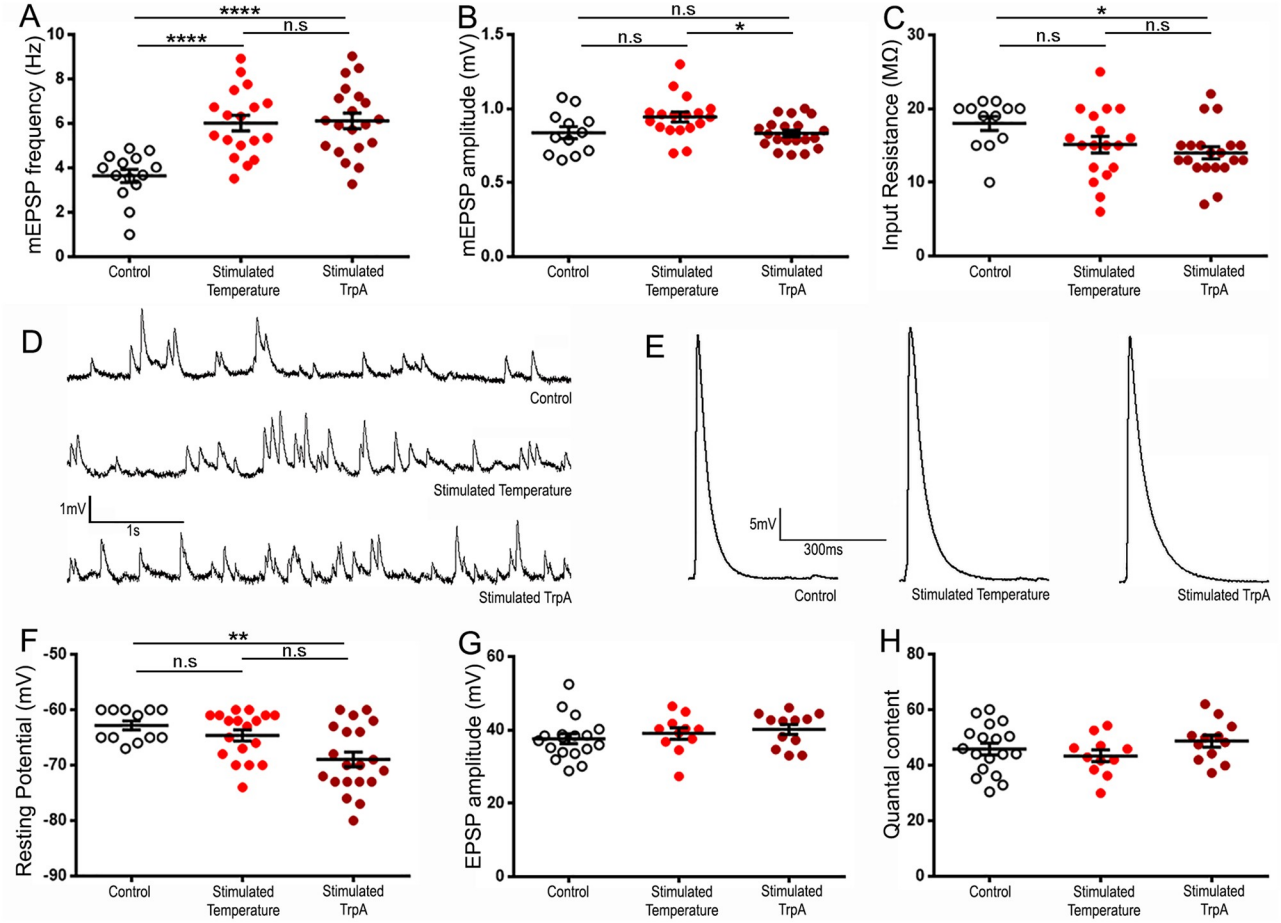
Temperature and TRPA-driven activity-dependent electrophysiological plasticity at the *Drosophila* NMJ. A: Quantification of the average mEPSP frequency with and without stimulation. B: Quantification of the average mEPSP amplitude with and without stimulation. C: Quantification of the average muscle input resistance with and without stimulation. D: Representative electrophysiological recordings showing spontaneous mEPSP in control and stimulated preparations. E: Representative electrophysiological recordings of evoked EPSPs in unstimulated and stimulated preparations. F: Quantification of the average muscle resting potential with and without stimulation. G: Quantification of the average evoked EPSP amplitude in unstimulated and stimulated preparations H: Quantification of the average quantal content (number of vesicles released by action potential). Animals were D42-Gal4/+ (Control and Stimulated temperature) and UAS-TrpA/+; D42-Gal4/+ (Stimulated TrpA). **** is p < 0.0001; ** is p < 0.01; * is p < 0.05. A one-way ANOVA with Holm-Sidak’s multiple comparisons test was applied in A, B, G, H. A Kruskal-Wallis analysis with Dunn’s post-test was performed in C and F. There were no significant differences detected by this test in G and H. All quantifications show SEM. Scale is 10 μm.

Intrigued by the ability of temperature and patterned TRPA-driven stimulation to evoke activity-dependent synaptic plasticity at the morphological level, we asked whether these conditions could drive electrophysiological changes. We focused on the stimuli that consist of patterned pulses of temperature from 23°C to 27°C, as being the smallest temperature fluctuation capable of activating and inactivating TRPA channels. We first wondered about the physiological status of the preparations after exposure to pulses of temperatures. We found that the input resistance of the preparations submitted to 23°C to 27°C temperature pulses (average of 15.1 ± 1.1 MΩ; Fig 5C) was no different from controls that were not exposed to pulses of temperatures (18 ± 1 MΩ; Fig 5C; p = 0.17). The stimulated preparations containing the TRPA transgene showed a slight decrease in input resistance compared to controls (14 ± 0.8 MΩ; Fig 5C; p = 0.013), but this decrease was much subtler than the one observed with the K^+^ depolarization protocol (Stimulated 120 min showed 6.8 ± 0.9 MΩ; Fig 1G). In addition, an input resistance of 14 MΩ is still considered to indicate a healthy preparation. When we looked at the resting potential of these preparations, we found that TRPA-driven stimulated preparations showed resting potentials more hyperpolarized than control preparations (-69 ± 1.3 mV compared to -62.8 ± 0.8 mV; Fig 5F; p = 0.007) while temperature alone was as hyperpolarized as controls (-64.6 ± 1 mV; p = 0.76). Together these results suggest that the preparations are healthy following such stimulations. This gave us the opportunity to ask whether mEPSP frequency and amplitude as well as EPSP amplitude could be affected by such a treatment. We first asked whether temperature alone or temperature-triggered TRPA opening could provoke an increase in mEPSP frequency after patterned stimulation. We find that, as with the morphological modifications, patterned temperature pulses alone were sufficient to elicit electrophysiological changes typical of activity-dependent synaptic plasticity. Indeed, while the mEPSP frequency of control preparations always kept at 20°C is 3.6 ± 0.3 Hz on average, it is 6 ± 0.4 Hz for preparations subjected to patterned pulses of temperature from 23°C to 27°C (Fig 5A and 5D; p = 0.0002). Surprisingly, and in contrast to what we observed for morphological modifications, there is no added effect of TRPA-driven depolarization on the frequency of mEPSPs (6.1 ± 0.3 Hz; Fig 5A and 5D; p > 0.99). Regarding the mEPSP amplitudes, we found that none of the stimuli (stimulated temperature 0.94 ± 0.03 mV and stimulated TrpA 0.83 ± 0.02 mV) can elicit a statistically significant increase in the average of mEPSP amplitudes (control 0.84 ± 0.04 mV; Fig 5B and 5D; p = 0.055 and p = 0.91 respectively) in contrast to what we observed with optogenetics (Fig 2E and 2F) and the High K+ protocol (Fig 1E and 1F). This suggests that the use of TRPA warmth-gated channels and exposure of transgenic larva to different temperatures might not evoke the same response than the one we observed with optogenetics. In these experiments, we did not observe any changes in the evoked response or the quantal content after temperature- (EPSP amplitude = 39.1 ± 1.6 mV compared to 37.6 ± 1.4 mV in control; p = 0.72; and QC = 43.4 ± 2.1 compared to QC = 45.8 ± 2.1 in control; p = 0.56) or TRPA-driven stimulation (EPSP amplitude = 40.2 ± 1.4 mV; p = 0.49; and QC = 48.7 ± 2.1; p = 0.56; Fig 5E, 5G and 5H).

## Discussion

The *Drosophila* NMJ is a major model for studying basic phenomena underlying synaptic growth and function. Because *Drosophila* research has access to numerous genetics strategies, many experimental avenues exist for eliciting activity-dependent synaptic plasticity and assessing its mechanisms. Here we focused on three different strategies to depolarize experimental preparations: the addition of a potassium-rich depolarizing solution, a transgenically-encoded depolarizing light-sensitive cation channel (CsChrimson), and a transgenically-encoded depolarizing warmth-sensitive cation channel (TRPA1). In addition, we contrasted the use of continuous stimulation with patterned stimulation protocols. Continuous activation of neurons has been achieved successfully with optogenetics. In the adult fly central nervous system, optogenetics using the red-activatable Channelrhodopsin ReaChR showed spiking activity decays during continuous light stimulation [56]. At the larval NMJ, optogenetics has been used for acute and chronic activation of the Channelrhodopsin variant ChR2-XXL with blue light pulses that ranged from 10 seconds to 1 hour of constant light exposure [67]. The same is true for TRPA [33]. Ectopic expression of TRPA channels in R8 photoreceptor cells of adult flies allowed for the persistent activation of photoreceptors that extended for days [35]. An earlier study expressed TRPA channels in circadian neurons of adult flies and achieved continuous activation of these neurons by exposing flies to 27°C [68]. At the NMJ, continuous TRPA stimulation for 1 hour can provoke morphological changes at muscle 4 when driving UAS-TRPA1 construct with the VGlut/OK371gal4 [69] motoneuron driver [30]. But the search for a stimulus that better resembles the physiological situation has resulted in labs developing patterned stimulation protocols, which alternate periods of activity with periods of rest [23]. Indeed, the patterned stimulation protocols used at the *Drosophila* NMJ are similar to the protocols of spaced depolarizations used to promote structural plasticity in dendritic spines of hippocampal neurons in culture [42]. In the present study we also allowed the preparations different rest durations; we examined them 15 or 45 minutes after the last stimulation to assure that these modifications were lasting effects, a condition *sine qua non* of activity-dependent synaptic plasticity [6].

The stimulation settings used in this work are very diverse and might correspond to different experimental needs. The ease of use is an important factor when considering which technique to select. The potassium rich protocol complies fully with this parameter. Because it does not use transgenes, it is very accessible and very attractive to laboratories working with undergraduate student scientists. Nonetheless, in our experience, a successful High K+ stimulation protocol is achieved only after significant training of the researcher. In addition, many experimental questions will require genetic backgrounds containing specific alleles and/or expressing transgenes. The addition of more transgenes solely designed to depolarize the preparation can be challenging even to the experienced geneticist. Nevertheless, our present results indicate that depolarizing the preparations using the potassium-rich protocol limits drastically any electrophysiological work attempting to measure mEPSP or EPSP amplitudes. Indeed, after this treatment, the preparations show smaller input resistance and depolarized resting potential. Because we did not observe similar events with the stimulating protocols using temperature, temperature driven TRPA stimulation or optogenetics, we strongly favor the hypothesis positing that K+ stimulation has a deleterious effect on the muscle. Nevertheless, it is possible that the changes in membrane properties could also be part of the plasticity and/or a compensation to this plasticity. Indeed, after heat treatment [70] or during repetitive synaptic activity (train of electrical stimulation at 20Hz; [71]), a decrease in input resistance has been observed. In addition, at high temperature rearing, synaptic homeostasis takes place to maintain a normal EJP within a terminal that contains increased release sites [72]. This is achieved by decreasing quantal size through a decrease of muscle input resistance. In any case, the changes in muscle input resistance and resting potential do not pose a direct limitation for assessing differences in mEPSP frequency, although it may result in an underestimation caused by missing smaller events during quantification. Another way to minimize the effect on input resistance and membrane potential might be the use of direct electrical stimulation [23, 29]. It is still an invasive preparation, but it utilizes a much more physiological stimulation paradigm.

Another parameter to take into consideration is the potency/scale of the response. Indeed, not all the methods seem to provoke the same number of morphological changes. A patterned stimulation using the potassium-rich depolarizing solution appears to provoke the most important synaptic remodeling (7.9 boutons per synapse) while a more modest response was observed with optogenetics (4.7 boutons per synapse). The temperature-driven response and the temperature-triggered TRPA also provoked comparable activity-dependent synaptic plasticity morphological changes (4.4 boutons per synapse and 7.6 boutons per synapse, respectively).

In addition to the robustness of the response, the specificity of the stimulus should also be considered. Using optogenetics to produce motoneuron-only patterned stimulation seems to be the most specific manner to elicit activity-dependent synaptic plasticity. Indeed, our experiments show that control preparations do not show morphological changes induced by the culture conditions (raised in the dark in presence of all-trans retinal containing food) nor the stimulation protocol (patterned flashes of red light). We can conclude that the synaptic changes that we observed are only due to the depolarization of the motoneuron and the resultant presynaptic neurotransmitter release at the NMJ. Surprisingly, the efficacy of the potassium depolarization protocol also shows a requirement for presynaptic stimulation. The requirement for presynaptic release to elicit morphological changes after repeated stimulation has been established [21–23, 27]. When presynaptic release is compromised by perturbing action potential formation (by using a Na+ channel mutant; para^ts^) or presynaptic vesicle fusion (by using synaptotagmin 1 mutants; a fast mediator of neurotransmitter vesicle release and presynaptic Ca^2+^ sensor), ghost bouton formation after repeated potassium-driven stimulation is prevented. This suggests that the source of depolarization that elicits these persistent morphological changes is presynaptic. Nevertheless, it is also known that such potassium shocks can depolarize the terminal directly both pre-and postsynaptically [73]. Such a generalized effect might apply to the temperature-evoked stimulation. Indeed, we showed that temperature alone can elicit morphological and electrophysiological changes. It is, at this stage, not possible to assess whether this phenomenon is due to an effect of the temperature on neurons or muscles or both. We also showed that driving TRPA opening in neurons can increase the morphological changes at the synapse when compared to temperature only stimulation, suggesting that presynaptic only stimulation can provoke an additional effect on morphological changes. It is possible that increased locomotory activity within the experimental setup (thermocycler, see Materials and methods) accounts for some of the effects described here. Rapid temperature changes increase nociceptive rolling behavior of larva [74] and increased locomotion can elicit morphological changes at the NMJ [29, 74, 75]. In contrast, we show that driving TRPA opening in motoneurons does not further increase activity-dependent potentiation of spontaneous neurotransmission when compared to temperature only stimulation, suggesting temperature alone accounts for all electrophysiological changes described.

This demonstration of the effects of temperature on a nerve terminal is quite remarkable and the first such characterization at the *Drosophila* NMJ. It is a consistent result that we can observe with a small temperature increase (4°C difference, 23°C to 27°C applied in a patterned manner). A number of studies have pointed out the consequences of temperature on the nervous system [76, 77]. For example, numerous studies provide evidence that the properties of neurotransmission vary depending on the temperature of the synapse. Interestingly, within the mammalian brain, each brain structure has its own basal temperature, and the subtle differences in temperature persist even when the environmental conditions impose drastic changes in absolute temperatures [76]. *In vivo* experiments on the mammalian neocortex demonstrate that neuronal activity changes in response to temperature. When brain temperature is decreased, pyramidal neurons from layer 2/3 of the neocortex are depolarized and their input resistance increases [78]. This was also found *in vitro* using acute slices [79]. Using rat hippocampal slices, the effect of temperature in evoked neurotransmission was shown to modify the presynaptic compartment by affecting the amount of vesicles released [80]. At the calyx of Held, temperature is also capable of modifying the dynamics of exocytosis [81]. Interestingly, and relevant to our study, acute temperature shifts can modulate short term synaptic plasticity properties in hippocampal cell cultures. *In vitro* experiments on rat hippocampal synapses demonstrated that temperature affects the properties of short-term plasticity [82]. In CA1 pyramidal neurons, constant trains of stimulation at a frequency of 40 Hz at 23°C vs 33°C showed that temperature promotes changes in evoked field EPSPs whereby synapses display depression at 23°C and potentiation over 33°C [82].

In this study we have presented different techniques for eliciting activity-dependent synaptic plasticity and described their different characteristics. While a subset allows meaningful electrophysiological assessment, all of them show morphological modifications. It has been previously shown that the ghost boutons could be heterogenous [29]. Indeed, intense activity promotes the rapid appearance of new synaptic boutons, some filled with synaptic vesicles possibly capable of exocytosis/endocytosis while other ghost boutons lack synaptic vesicles but contain filamentous matrix and membrane folds. Interestingly, both types of ghost boutons persist and remain unchanged for at least 60 minutes of rest after the last stimulation [29]. It was previously reported that some ghost boutons are evident within seconds of the first cycle of stimulation [21], suggesting some ghost boutons appear from primed synapses ready to respond to increased activity while others appear later, only after subsequent pulses of patterned depolarizations. Others have described that lasting structural changes appear after the 4^th^ cycle of repeated stimulation [23]. It remains to be determined whether the different stimulation techniques described here evoke homogeneous or heterogeneous sets of activity-dependent changes.

## Supporting information

S1 Table Electrophysiological recordings of the muscle input resistance (IR) and resting potential (RP) in unstimulated controls and K^+^ stimulated preparations. The 16 control preparations have an IR ≥ 5 MΩ and RP ≤ -60 mv. In contrast, 14 stimulated preparations (marked with grey background) out of 22 (64%) failed to meet these standards.
(TIF)

S1 Data (XLSX)
