# Supplementary figures and images for: A comparison of three different methods of eliciting rapid activity-dependent synaptic plasticity at the Drosophila NMJ
Source: PLoS One. 2021 Nov 30;16(11):e0260553. doi: 10.1371/journal.pone.0260553 (PMC8631638; doi:10.1371/journal.pone.0260553)

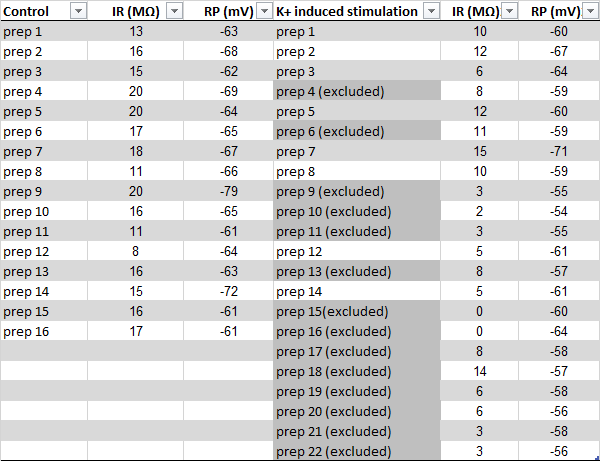

Supplement: S1 Table — The 16 control preparations have an IR ≥ 5 MΩ and RP ≤ -60 mv. In contrast, 14 stimulated preparations (marked with grey background) out of 22 (64%) failed to meet these standards. (TIF) [file pone.0260553.s001.tif]
